# A Service Evaluation of More Than 27 000 Adults Referred to a Community Weight Management Program: 24-Month Outcomes

**DOI:** 10.1177/21501319241291784

**Published:** 2024-10-21

**Authors:** Josef Toon, Sarah-Elizabeth Bennett, Jacquie Lavin, Carolyn Pallister, Amanda Avery

**Affiliations:** 1 Slimming World, Derbys, UK; 2 De Montfort University, Leicester, UK; 3 University of Nottingham, Nottingham, UK

**Keywords:** obesity, weight loss, weight loss maintenance, referral, BMI, socioeconomic status

## Abstract

**Background:**

Obesity has a significant impact on healthcare resources with limited accessible support available through the NHS. This service evaluation determines 24-month efficacy of referral to an open-group behavioral program by BMI category and socioeconomic status.

**Methods:**

This retrospective, longitudinal study examined weight outcomes of adults living in England referred by healthcare professionals to Slimming World during 2016 who recorded at least 1 weight change. Primary outcome was % weight change at 3, 6, 12, and 24 months. Socioeconomic status was measured using the Index of Multiple Deprivation (IMD). Data from a post-referral questionnaire investigated self-reported changes in dietary and activity behaviors.

**Results:**

Twenty-seven thousand five hundred sixty (15.6% male) records were analyzed. Mean (SD) age and BMI on joining were 48.6 (14.80) years and 37.1 (6.31) kg/m^2^; 91.7% had a BMI > 30 kg/m^2^. Mean (SD) % weight change was −5.6 (3.79), −7.1 (5.71), −7.5 (6.88), and −7.3 (6.88) at 3, 6, 12, and 24-months, respectively. At 24- months, differences in weight loss between BMI category were significant, ranging from 0.29% (35-<40 vs 40+) to 1.33% (25-<30 vs 40+). For IMD quintile only comparisons against Q1 and Q2 were significant, ranging between 0.36% (Q2 vs Q3) to 0.94% (Q1 vs Q5). Five thousand eight hundred sixty-two (21.2%) completed the post-referral questionnaire. There were no BMI category effects on dietary behaviors but changes in physical activity behaviors were lower within the higher categories albeit effect sizes were small (all ges < 0.001). IMD quintile influenced changes for sugary drinks, watching TV and avoiding moderate activity although effect sizes were small (all ges < 0.01).

**Conclusion:**

Following 12-week referral to a commercial weight management organization, a mean weight loss of over 7% was reported at 24-months. Adults with higher BMIs and a greater level of deprivation can benefit from the practical support offered as part of the referral, supporting weight loss and weight loss maintenance albeit with some inequality.

## Introduction

Obesity is recognized as being a complex chronic condition with multi-factorial causes. People living with obesity face a future of living with long-term ill health given the strong association between obesity and most of the well-known non-communicable diseases. More severe obesity is associated with a greater level of health risk.^1^ Thus obesity, given the prevalence, poses a significant burden on health care resources and impacts the quality of care that current national health care systems can provide.^2^ Long-term support is required that is scalable and easily accessible by people from different backgrounds and with different levels of obesity to enable them to better manage their weight long-term. Despite the individual and societal costs associated with obesity, limited long-term support is available through the NHS.

The management of obesity needs behavioral solutions that are accessible to people within the community which can accommodate people’s daily routines.^3^ Accessibility includes the need to be able to equally support people from different socioeconomic backgrounds given that there are disparities in adult obesity prevalence. Data from the 2019 Health Survey for England shows 39.5% of women living in the most deprived areas of England are living with obesity compared to 22.4% women living in the least deprived areas. Comparable data for men is 30.2% versus 21.9%.^4^ The UK National Institute for Health and Care Excellence (NICE) recognizes the importance of providing regular and ongoing support for healthier behavioral change, to prevent weight gain, promote weight loss and its maintenance. NICE recognizes the supportive role of commercial weight management organizations (CWMOs) which follow guidance criteria for best practice and suggests that a 3% weight loss should be achievable following a 12-week intervention.^5^

UK weight management organization, Slimming World, pioneered “referral” schemes in 2000 and were the first CWMO to actively support the building of partnerships with the NHS. A feasibility study^6^ showed that referral to the CWMO can offer an effective solution to reduce overweight and obesity levels in the community. The pilot scheme provided recognition of service quality, patient satisfaction, and value for money and the model is now well-established. Practitioners can refer patients to the CWMO, offering them free membership and attendance at a local community group of their choice, ordinarily in blocks of 12 weeks. The cost is funded by Local Authorities or Clinical Commissioning Groups (CCGs) and subsidized by the CWMO, making this a very cost-effective intervention^7^ and helping to reduce inequalities for those people who may not be able to self-fund.

Primary care referral to an open-group behavioral program has been shown to be an effective and healthy strategy for the management of obesity,^8,9^ but there is limited data showing 24-month weight outcomes for adults referred for a 12-week program who have different levels of obesity and come from different backgrounds as determined by Index of Multiple Deprivation (IMD). Two of the 5 recommendations for future weight management research in the UK includes reviewing the effect of weight management lifestyle programs on changes to dietary habits, physical activity levels, and sedentary behavior while also considering the impact of short-term interventions on longer term outcomes.^10^ Thus, this service evaluation aims to determine the 24-month efficacy of Slimming World on Referral, reporting on all adults meeting inclusion criteria who were referred during 2016.

## Methods

This retrospective, longitudinal study examined weight outcomes in adults referred to the Slimming World program during 2016. The data analysis was categorized as a service evaluation, designed to answer the question “What standard does this service achieve?,” in accordance with the Health Research Authority’s^11^ definitions of service evaluation, clinical audits and usual practice.

## Participants

Participants were referred by a healthcare professional from 76 different schemes across England.

## Intervention

Participants attended 1 of the 19 000+ community-based groups of their choice and were able to attend 12 weekly sessions at no cost to themselves. The program uses a range of evidenced-based behavior change techniques with members receiving support from trained group facilitators. A variety of tools are available to help members develop and maintain long-term healthier behaviors to maintain weight loss. For example, self-regulation strategies are encouraged through weekly recording of weight, the option to use food and activity diaries to monitor and reflect on food intake and physical activity levels, and individual motivation and peer support to help participants become more confident in changing their mindset and to develop action plans and goal setting (https://www.slimmingworld.co.uk/how-it-works). Additional online support is available to complement the weekly group-based support. Participants had the option to continue attending the open-ended group at their own cost.

## Design

The primary outcome was percentage weight change from baseline measured at 3 months (12 weeks), 6 months, 12 months, and 24 months. Missing data was imputed using last observation carried forward (LOCF), in which the latest available weight measurement is used preceding the given time point.

All data was obtained electronically from Slimming World’s securely stored reporting database using Microsoft Structure Query Language. This extraction was part of a routine, provider-led audit of outcomes for those joining the service during 2016. Extracted data included joining demographics such as age and gender along with joining weight and height to derive BMI and weekly weight data, which was captured using standardized, calibrated scales.

Socioeconomic status was measured using the Index of Multiple Deprivation (IMD) which classifies the relative deprivation of small geographic areas within England. Areas can be categorized into quintiles with quintile 1 denoting the 20% most deprived areas and quintile 5 the 20% least deprived. Most recent postcode data at the time of extraction was matched to an IMD decile. The assumption was made that it would be unlikely for people to move area during the 12-week referral period although members are able to change groups if they wish to.

The inclusion criteria for the service evaluation were defined a priori before commencing analysis and records were screened for accuracy. Any erroneous data or records that did not meet inclusion criteria were removed. Participant records were included if they/their:

- joined the program via referral from primary care and had a minimum of 1 reported weight change
- were aged between 18 and 80 years
- start weight was between 80 and 600 lbs (data is electronically reported in lbs)
- had a height between 1.35 and 2.10 m
- starting BMI was between 20 and 90 kg/m^2^
- were not pregnant, breastfeeding or had an eating disorder
- first week weight change was between ±10% and average weight change over subsequent weeks was between ±5% (data that would be within reasonable physiological parameters for changes in energy balance)
- had a valid postcode

Data were also extracted for the sample completing the optional post-referral questionnaire, which asked how participants felt about themselves before being referred compared to after the 12-week program. Self-reported changes in dietary and physical activity behaviors were asked using Likert 5-point scales (1 = *strongly disagree* and 5 = *strongly agree*).

## Statistical Analysis

All data and statistical analysis were conducted using R with data described using descriptive and inferential statistics. Data were analyzed using mixed ANOVA to compare the weight outcomes across time points by BMI category and IMD quintile. Post-hoc comparisons were performed using Tukey’s tests.

## Ethical Considerations

Upon joining the program participants are informed of the data collection process and provide consent for their anonymized data to be used for research and evaluation purposes. Further details on the data protection policy can be found at the following link: https://www.slimmingworld.co.uk/privacy-policy.

**Figure 1.**
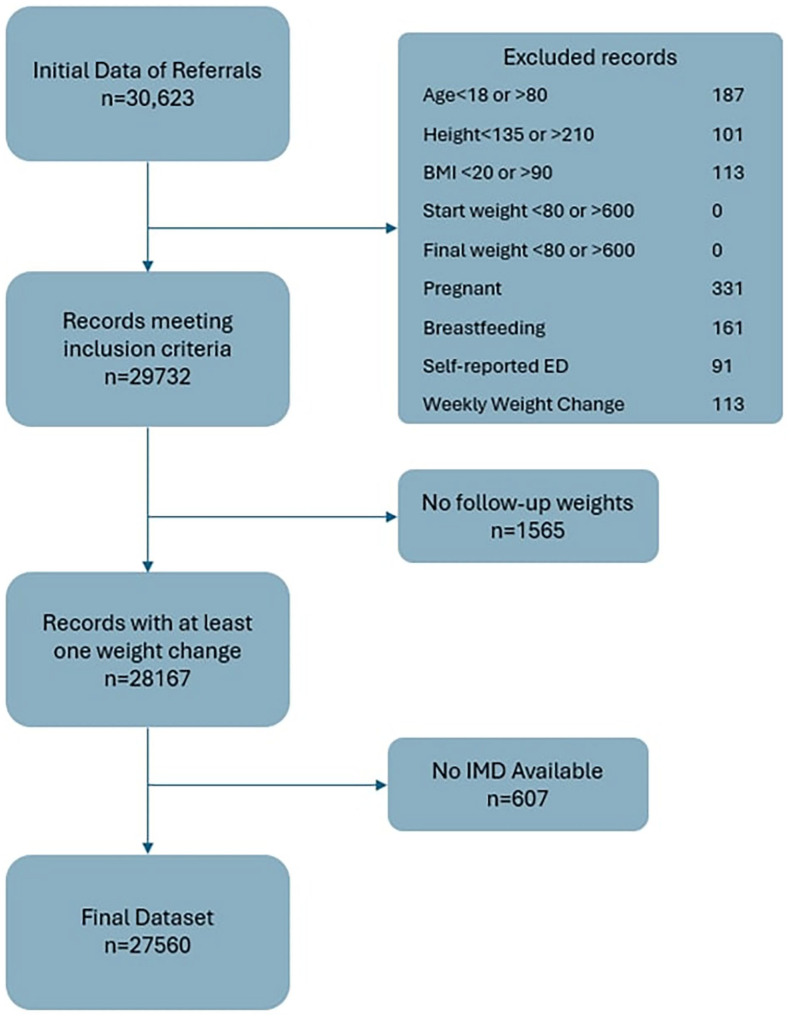
Inclusion and exclusion criteria flow diagram.

After exclusions, 27 560 patient records were included for analysis. Figure 1 below shows inclusion and exclusion criteria adapted from the CONSORT flow diagram.

## Baseline Demographics

Of the sample, 15.6% of referral patients were male (Table 1). Mean (SD) age on joining the program was 48.6 (14.80) years. Mean (SD) BMI on joining the program was 37.1 (6.31) kg/m^2^ with 91.7% of adults referred having a joining BMI greater than 30 kg/m^2^, 64.6% with a joining BMI between 30 to <40 kg/m^2^ and 27.1% with a BMI of 40 kg/m^2^ or greater and classed as living with severe obesity. In total, 15.0% were living within Q1, 18.5% were in Q2, 23.1% were in Q3, 21.6% were in Q4, and 21.9% were in Q5 as determined by the IMD data on joining.

**Table 1.** Baseline Demographics of Females and Males.

	Female (n = 23 272)		Male (n = 4288)		*z*	*P*	*r*
	*M*	SD	*M*	SD			
Age	47.8	14.79	53.1	14.02	−8.31	<.001	.05
Joining BMI	37.0	6.32	37.8	6.23	−21.62	<.001	.13

## Weight Outcomes

Mean (SD) weight change was −5.62 (3.79)% at 12-weeks, −7.07 (5.71)% at 6 months, −7.52 (6.88)% at 12 months, and −7.30 (6.88)% at 24 months. After the 12-week intervention, 72.1% of the participants had achieved a weight loss of ≥3%, 53.7% had achieved a weight loss of ≥5%, and 13.2% achieved a weight loss of ≥10%. Table 2 shows the weight loss at each time point for the whole dataset, by BMI category and IMD quintile.

**Table 2.** Means (*M*) and Standard Deviations (SD) of Percentage (%) Weight Change by Time Point, BMI Category, and IMD Quintile.

		12 Weeks		6 Months		12 Months		24 Months	
		*M* (%)	SD	*M* (%)	SD	*M* (%)	SD	*M* (%)	SD
All		−5.62	3.79	−7.07	5.71	−7.52	6.88	−7.30	6.88
BMI category	25 to <30	−5.46	3.69	−6.54	5.10	−6.60	5.43	−6.35	5.25
	30 to <35	−5.74	3.81	−7.12	5.59	−7.40	6.35	−7.13	6.19
	35 to <40	−5.63	3.81	−7.11	5.81	−7.62	7.07	−7.40	7.06
	40+	−5.51	3.75	−7.13	5.93	−7.84	7.67	−7.69	7.99
IMD quintile	Q1	−5.21	3.76	−6.49	5.57	−6.87	6.68	−6.67	6.75
	Q2	−5.42	3.85	−6.78	5.71	−7.21	6.79	−7.01	6.87
	Q3	−5.64	3.84	−7.09	5.81	−7.57	7.02	−7.36	7.04
	Q4	−5.80	3.74	−7.34	5.70	−7.79	6.90	−7.59	6.99
	Q5	−5.88	3.70	−7.42	5.68	−7.89	6.90	−7.61	6.85

In total, 64.5% (n = 17 765) continued attending after their 12-week referral and on average attended a further 16.4 (SD = 13.8) sessions. Of this subset, 29.2% (n = 5191) were high attenders at 12 months, (attending ≥41 sessions) and had a mean (SD) weight change of −15.3% (7.93%).

The analysis of weight change (Table 3) revealed statistically significant but marginal 2-way interactions between BMI category and time point, *F*(4.90,44 998.75) = 55.02, *P* < .001 and IMD quintile and time, *F*(6.54,44 998.75) = 3.56, *P* < .001.

**Table 3.** Mixed ANOVA of Weight Change by Time Point, BMI Category, and IMD Quintile.

Effect	DFn	DFd	*F*	*P*	ges
BMI category	3.00	27 540	13.34	<.001	<0.001
IMD quintile	4.00	27 540	15.88	<.001	0.002
Time	1.63	44 998.75	2164.86	<.001	0.009
BMI category : IMD quintile	12.00	27 540	0.63	.82	<0.001
BMI category : Time	4.90	**44** 998.75	55.02	<.001	<0.001
IMD quintile : Time	6.54	**44** 998.75	3.56	<.001	<0.001
BMI category : IMD quintile : Time	19.61	44 998.75	0.38	0.99	<0.001

Separate follow-up main effects analyses for these 2-way interactions revealed significant but marginal differences across time points (all *P* < .001). The estimated mean differences and 95% confidence intervals of the post-hoc Tukey’s tests are reported in Table 4.

**Table 4.** Estimated Mean Differences and 95% Confidence Intervals of Weight Change Across Time by BMI Group Category and IMD Quintile.

Group 1	Group 2	12 Weeks	6 Months	12 Months	24 Months
25 to <30	30 to <35	−0.29 [−0.51,−0.06]	−0.58 [−0.93,−0.24]	−0.8 [−1.21,−0.39]	−0.77 [−1.18,−0.36]
25 to <30	35 to <40	ns	−0.57 [−0.92,−0.22]	−1.02 [−1.43,−0.6]	−1.05 [−1.47,−0.63]
25 to <30	40+	ns	−0.59 [−0.94,−0.24]	−1.24 [−1.66,−0.81]	−1.33 [−1.76,−0.91]
30 to <35	35 to <40	ns	ns	ns	−0.28 [−0.54,−0.01]
30 to <35	40+	0.24 [0.09,0.39]	ns	−0.44 [−0.71,−0.17]	−0.56 [−0.84,−0.29]
35 to <40	40+	ns	ns	ns	−0.29 [−0.57,−0.02]
Q1	Q2	ns	ns	ns	ns
Q1	Q3	−0.43 [−0.63,−0.22]	−0.61 [−0.92,−0.3]	−0.71 [−1.08,−0.33]	−0.69 [−1.07,−0.32]
Q1	Q4	−0.59 [−0.8,−0.38]	−0.85 [−1.16,−0.53]	−0.92 [−1.3,−0.54]	−0.93 [−1.31,−0.54]
Q1	Q5	−0.66 [−0.87,−0.46]	−0.93 [−1.25,−0.62]	−1.02 [−1.4,−0.64]	−0.94 [−1.32,−0.56]
Q2	Q3	−0.22 [−0.41,−0.02]	−0.31 [−0.6,−0.02]	−0.37 [−0.72,−0.01]	−0.36 [−0.71,−0.01]
Q2	Q4	−0.38 [−0.58,−0.18]	−0.55 [−0.85,−0.25]	−0.58 [−0.94,−0.22]	−0.59 [−0.95,−0.23]
Q2	Q5	−0.45 [−0.65,−0.26]	−0.64 [−0.93,−0.34]	−0.68 [−1.04,−0.32]	−0.6 [−0.96,−0.25]
Q3	Q4	ns	ns	ns	ns
Q3	Q5	−0.24 [−0.42,−0.05]	−0.33 [−0.61,−0.05]	ns	ns
Q4	Q5	ns	ns	ns	ns

Abbreviation: ns, not significant.

At 12 weeks, only comparisons between the 25 to <30 and 30 to <35 BMI group and the 30 to <35 and 40+ group were significant. For IMD quintile all but 3 comparisons (Q1 vs Q2, Q3 vs Q4, and Q4 vs Q5) were significant.

At 6 months, only the comparisons against the 25 to <30 BMI group were significant. For IMD quintile all but 2 comparisons (Q3 vs Q4 and Q4 vs Q5) were significant.

At 12 months, all comparisons against the 25 to <30 BMI category were significant as were the comparisons between the 30 to <35 and 35 to <40 group. For IMD quintile only comparisons against Q1 and Q2 were significant.

At 24 months, all comparisons for BMI category were significantly different, with differences in weight change ranging between 0.29% (35-<40 vs 40+) to 1.33% (25-<30 vs 40+). For IMD quintile, significance and magnitude of differences between quintiles were similar to those observed at 12 months.

## Evaluation Questionnaire Analysis

In total, 21.2% of participants completed the post-referral questionnaire. Those completing the questionnaire were older (*M* = 51.2 years, SD = 13.7 years) than non-completers (*M* = 47.9 years, SD = 15.0 years, *z* = −15.1, *P* < .001) but they did not differ on joining BMI. Weight loss at the end of the referral was greater for survey completers (*M* = −7.08%, SD = 3.62%) compared to non-completers (*M* = −5.23%, SD = 3.73%, *z* = −34.0, *P* < .001).

Whether participants had improvements in the way they felt about themselves following referral were determined using a 5-point agreement scale (5 = *Agree very much*, 1 = *Do not agree*). Mean change score was 3.83 (SD = 1.22, Mdn = 4). There was no significant difference by joining BMI category or IMD quintile (both *P* > .05).

Mean scores of dietary and physical activity behaviors before and after the referral are reported in Table 5.

**Table 5.** Before and After Mean Scores and *t*-Test Differences for Reported Changes in Dietary Behaviors and Physical Activity.

	Before		After		*t*	*P*	*d*
	*M*	SD	*M*	SD			
I cook meals from scratch	3.22	1.37	4.23	1.16	54.23	<.001	0.71
I drink a lot of sugary drinks	1.84	1.28	1.13	0.54	−42.73	<.001	−0.56
I eat a lot of processed foods	2.77	1.36	1.41	0.82	−71.20	<.001	−0.93
I eat a lot of take-aways and fast food	2.40	1.34	1.23	0.65	−66.64	<.001	−0.87
I eat a lot of unhealthy snacks at work and home	3.18	1.38	1.29	0.74	−96.61	<.001	−1.27
I eat 5 portions of fruit and vegetables per day	2.82	1.37	4.18	1.17	67.65	<.001	0.89
I do a lot of physical work around the house and garden	2.55	1.24	3.20	1.23	45.17	<.001	0.52
I go out for walks a lot	2.39	1.27	3.32	1.28	57.90	<.001	0.73
I play a lot of sports and go to the gym a lot	1.62	1.03	2.00	1.31	26.69	<.001	0.32
I tend to avoid intense activity	2.85	1.46	2.17	1.23	−36.77	<.001	−0.50
I tend to avoid moderate activity	2.30	1.33	1.73	1.05	−33.29	<.001	−0.48
I watch TV a lot	3.24	1.30	2.53	1.10	−50.94	<.001	−0.59

Mixed ANOVA revealed no significant 3-way interaction between time, BMI category and IMD quintile and no 2-way interactions between BMI category and IMD quintile. All significant 2-way interactions between BMI category and time and IMD quintile were followed-up by comparing before and after scores separately as seen in Table 6.

**Table 6.** Follow-Up ANOVA Comparing Changes in Dietary Behaviors Before and After Referral by BMI Category and IMD Quintile.

	Before			After		
	*F*	*P*	*ges*	*F*	*P*	*ges*
BMI category						
Cook meals from scratch	12.18	*	0.006	1.13	ns	0.001
Sugary drinks	16.16	*	0.008	0.42	ns	0.000
Processed food	34.06	*	0.017	0.60	ns	0.000
Take aways	50.04	*	0.025	0.64	ns	0.000
Unhealthy snack food	27.22	*	0.014	0.67	ns	0.000
Fruit and veg	9.71	*	0.005	0.92	ns	0.000
Go out for walks	49.55	*	0.025	19.93	*	0.010
Avoid intense activity	35.05	*	0.018	6.57	*	0.003
Avoid moderate activity	53.39	*	0.027	13.14	*	0.007
Watch a lot of TV	22.10	*	0.011	6.49	*	0.003
Physical work around house	38.84	*	0.020	10.85	*	0.006
IMD quintile						
Cook meals from scratch	3.52	*	0.002	0.68	ns	0.000
Sugary drinks	10.16	*	0.007	2.82	*	0.002
Processed food	8.30	*	0.006	1.82	ns	0.001
Take aways	12.71	*	0.009	2.35	ns	0.002
Unhealthy snack food	4.55	*	0.003	0.74	ns	0.001
Fruit and veg	3.01	*	0.002	2.29	ns	0.002
Go out for walks	1.28	*	<0.001	1.60	ns	0.001

Abbreviation: ns, not significant.

* *P* < .05.

**Figure 2.**
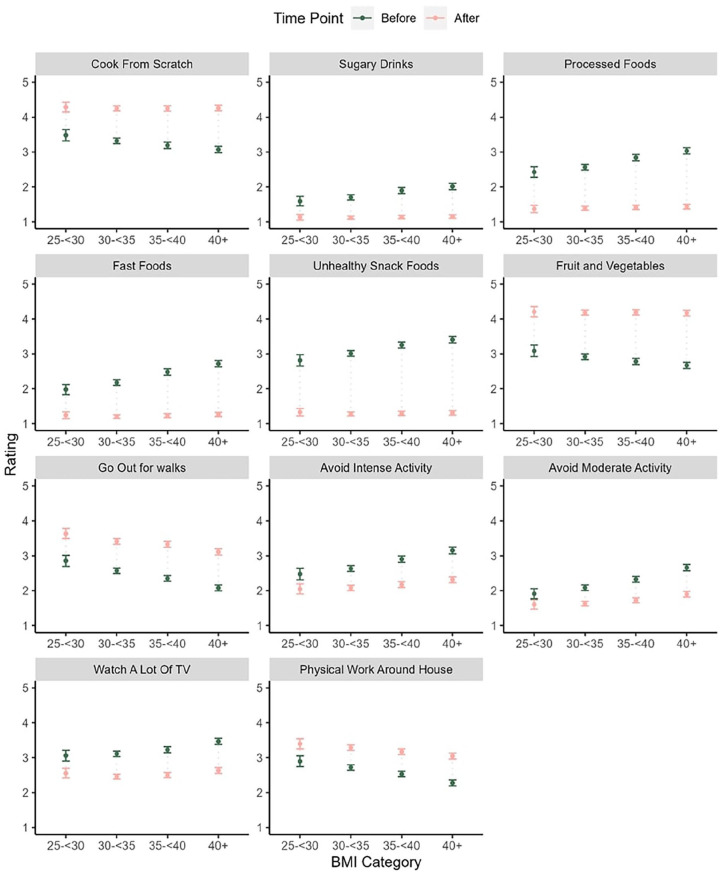
Mean and 95% CI for dietary and activity behavior ratings before and after referral by BMI category.

Mean scores by BMI category are shown in Figure 2.

The general pattern of the before scores for dietary behaviors was that higher BMI categories had higher mean scores on processed food, snack food, and sugary drinks consumption and lower scores on fruit and vegetable consumption and cooking from scratch. A similar pattern was observed for physical activity with lower before scores in activity engagement (eg, going out for walks) and higher avoidance scores (eg, avoiding moderate and intense activity) within the higher BMI categories. There were no significant effects on any of the dietary items for after scores but there were for the physical activity items. In general, changes were lower within the higher BMI categories although the magnitude of these effects was small (eg, all ges < 0.001)

**Figure 3.**
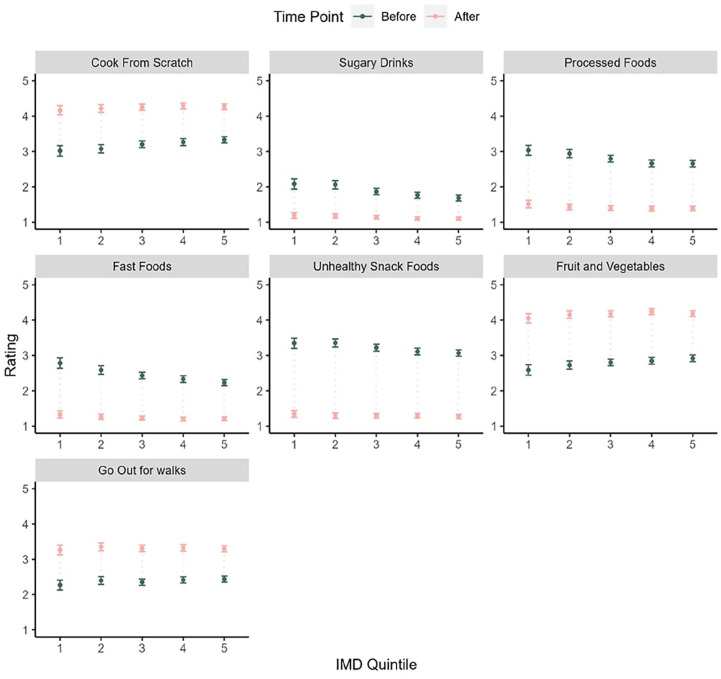
Mean and 95% CI for dietary and activity behavior ratings before and after referral by IMD quintile.

Mean scores by IMD quintile are shown in Figure 3.

For IMD quintile, before scores for the lower IMD quintiles were higher on processed food, snack food, sugary drinks, and fast-food consumption and lower on fruit and vegetable consumption and cooking meals from scratch and going out for walks. Differences in after scores for IMD quintile were only significant for sugary drink consumption although these differences were small (all ges < 0.01).

## Discussion

This service evaluation of over 27 000 adults referred from primary care to a community weight management (Slimming World) group during 2016 represents over 90% living with obesity and over a quarter living with severe obesity. The service evaluation includes data for over 4000 men and represents people across IMD quintiles.

All patients included in the evaluation had at least 1 weight change reported. More than half of the adults referred achieved a clinically significant weight loss of ≥5% after the 12-week intervention. The mean weight loss was almost 6% at 12 weeks, 7% at 6 months, 7.5% at 12 months with this loss maintained at 24 months. There were statistical differences between some of the time-points and levels of BMI category and IMD quintile, but these differences were marginal in terms of actual weight loss. Adults referred with a BMI of 40 kg/m^2^ or greater achieved a 7.7% weight loss at 12 months compared to 6.4% in those with an initial BMI of between 30 and 35 kg/m^2^. Those from the least disadvantaged quintile were able to achieve a 1% greater weight loss at 12 months compared to those from the most disadvantaged quintile. However, all mean weight losses were clinically significant.

These data compare favorably with findings from a previous study of 34 271 patients referred to Slimming World community groups which reported a mean weight loss of 4.0% at 12 weeks but did not monitor weight change for a longer period nor report on the influence of deprivation.^12^ We have also evaluated the effectiveness for participants self-selecting, rather than being referred, to join during 2016 across different socio-economic backgrounds.^13^ In this population of over 1 million adults, mean % weight change at 3, 6, and 12 months was 5.0 ± 3.6%, 5.9 ± 5.2%, and 6.0 ± 5.8%. Effect sizes comparing weight change between IMD deciles were negligible, with similar outcomes in the most and least deprived deciles at 12 months (5.7 ± 5.9% vs 6.2 ± 5.9%).^13^ It is noteworthy that, using this comparable data, adults who were referred to the same weight management program achieved slightly greater weight loss compared to those who chose to self-fund their attendance. This should be reassuring to referring commissioners and the healthcare practitioners making the referral as it suggests that people referred were equally, if not more so, committed to making behavior changes and benefitting from the support received from the community weight management program. A previous study found a mean weight loss of 7% in a group of women between 2.5 and 3 years after first joining a Slimming World group, regardless of whether they were still attending or not.^14^ Recent clinical trial data using the latest injectable pharmacotherapy reports a mean weight loss of 10% maintained at 4 years.^15^

Findings from our current evaluation shows that over 60% of those referred continued to access the support through self-funding and continued to attend for around a further 20 sessions which may have further contributed to the clinically significant weight loss at both 12 and 24 months. Whilst outside the scope of this current paper, it would be of interest to understand the sociodemographic characteristics of those who self-fund and continue to attend post-referral. Of those who continued attending, over 5000 were classed as high attenders, defined as attending 75% of the possible sessions, achieving a mean weight loss of over 15%.

There is limited data available from other providers of weight management services where open-group support is offered and the participants in these studies have a lower mean BMI at baseline. Ahern et al^16^ reported a mean weight loss of 3.2% at 12 weeks in 29 326 adults referred to WW (formerly Weight Watchers), over an 18-month period. The same authors then conducted a clinical trial where a 12-week referral resulted in a mean weight loss of 4.75 kg at 12 months which was greater than the loss resulting from a brief intervention.^8^ Follow-up at 5 years resulted in a weight loss of 1.95 kg which was perceived as being cost effective.^17^ Commercial provision of weight management in clinical studies has consistently been found to result in twice as much weight loss compared to standard care.^18^ Early outcome analysis of referrals to the English National Health Service Digital Weight Management Programme, where the intervention is delivered by a number of different providers, found non-significant differences in the weight change achieved between those referred from Q1 compared to Q5 but with a higher level of support offered for those referred from the more deprived areas. For those finishing the referral program, a mean weight change of −2.16 kg (95% CI −2.26, −2.05) for participants from Q1 and −2.36 kg (95% CI −2.48, −2.25) for participants from Q5 were reported.^19^

In this service evaluation, around a fifth of the patients completed the post-referral questionnaire and self-reported changes comparing status before the referral and after engaging with the Slimming World program. They reported feeling better about themselves and had made several changes to their dietary behaviors. These changes included cooking more meals from scratch, eating fewer processed foods, take-aways, and “fast” foods. They also had fewer unhealthy snacks at both home and work and fewer sugary drinks but were more likely to eat 5 portions of vegetables and fruit each day after they had been referred and engaged in the support offered. There were no differences in the magnitude of changes reported across BMI categories and the differences between IMD quintiles were marginal. There were differences in the “before” values, with those who had a higher BMI when referred and those who were from more disadvantaged locations, as determined by IMD, cooking fewer meals from scratch but having more processed, take-away and “fast” foods, more unhealthy snacks and sugary drinks although there was less difference in having 5 portions of vegetables and fruit before the referral.

Across the referred population, reported activity levels appeared to increase with reductions in sedentary behaviors through reduced time spent watching TV, going for more walks, and being more able to undertake housework. They were less likely to avoid moderate or intense activity and more likely to engage in sporting activities. For those with higher BMIs, they had lower change scores for engagement and higher scores for avoidance, and whilst small, the positive changes reported were less for those with higher BMIs. Across all areas of physical activity considered, changes did not appear to differ by IMD quintile except for going out for walks where those within the lower IMD quintiles showed a marginally smaller increase compared to the higher IMD quintiles.

For people to make and maintain healthier behaviors, it is important that they feel better about themselves. Research suggests that appropriate support needs to be offered in a sensitive and non-judgmental manner^20^ and that the guidance needs to be accessible to people from a range of backgrounds.^1^ Our research has consistently shown benefits to not just physical but also mental well-being with associated improvements in self-esteem.^21^ Eating fewer take-aways, “fast” and processed foods, unhealthy snacks and sugary drinks are likely be important dietary changes that will lead to overall health benefits besides supporting healthier long-term weight management. The health benefits of eating 5 portions of vegetables and fruit each day are well recognised^22^ and collectively the reported dietary changes, including cooking more meals from scratch, may help people managing on a low income to eat a nutritionally balanced healthier diet.^23^ It is well recognized that small but sustainable reductions in sedentary behaviors, alongside increased physical activity levels, will help support long-term weight loss maintenance.^1^

To the best of our knowledge, this is the first service evaluation reporting on 24-month outcomes for adults referred to a CWMO offering open-group community support. The efficacy has been reported for adults referred with different levels of obesity and across IMD quintiles, (at the time of referral), where it was found that all groups achieved clinically significant weight loss. Using LOCF data analysis has traditionally been seen to be best practice when evaluating weight management interventions^23^ although with limitations. Multiple data imputation (MI) and LOCF have resulted in similar differences between drug intervention and control being reported with the authors concluding that contrary to widely stated claims, LOCF did not produce a conservative efficacy estimate compared to MI. Also, LOCF resulted in a lower SE than MI.^24^ As this is a service evaluation of a real-life pragmatic intervention, it is inevitable that there will be cases of missing data which could arise systematically due to dropout from the program or non-systematically cases such as changes in personal circumstances, illness, or holiday absences from the program.

In this instance, we did not determine whether the adults referred had other co-existing comorbidities, but it is likely that many would have co-existing medical conditions such as type 2 diabetes and hypertension. Similarly, we did not determine if the members were taking any medication and, if so, whether their medications were reduced because of weight loss. Previous research suggests that people with co-morbidities are well supported with many needing less medication.^25^ Some of the referrers may have specifically targeted the opportunity to people with co-existing co-morbidities.

## Conclusion

For the 27 000+ women and men referred to Slimming World on Referral during 2016, a mean weight loss of over 7% was reported at 24 months. This weight loss represents a return on the investment of referral to a 12-week program albeit some choosing to self-fund and continue attending. The data shows equal efficacy for adults referred with different levels of obesity, including those living with severe obesity, and for people from different socioeconomic backgrounds. Similarly, the dietary and physical activity changes show very similar benefits to those with different levels of obesity and across the IMD quintiles. We can conclude that adults with higher BMIs and living on low incomes are able to benefit from the practical support offered as part of the 12-week referral program and are thus supported to lose and maintain their weight loss.
